# Evaluating the Impact of Filmarray Pneumonia Plus Panel in Therapeutic Decision-Making in Critical Patients with Suspected Respiratory Infection

**DOI:** 10.3390/antibiotics15050521

**Published:** 2026-05-21

**Authors:** Rosa Latorre Ibars, Sulamita Carvalho-Brugger, Paula Rodríguez Ibáñez, Montserrat Vallverdú Vidal, Silvia Iglesias Moles, Mar Miralbés Torner, Alba Bellés Bellés, Andrea Castellano, David Campi, Jesús Caballero, José Javier Trujillano Cabello

**Affiliations:** 1 Department of Intensive Care, Arnau de Vilanova University Hospital, 25198 Lleida, Spain; 2 Biomedical Research Institute (IRB) of Lleida, 25198 Lleida, Spain; 3 Department of Medicine, Universitat de Lleida (UdL), 25198 Lleida, Spain; 4 Department of Microbiology, Arnau de Vilanova University Hospital, 25198 Lleida, Spain; 5 Department of Intensive Care, Santa Maria University Hospital, 25198 Lleida, Spain

**Keywords:** multiplex PCR, FilmArray Pneumonia Panel, critical care, respiratory infection, antimicrobial stewardship

## Abstract

**Background:** Respiratory infections in critically ill patients remain a major challenge in intensive care units (ICUs), with high morbidity and mortality. Conventional microbiological methods often fail to identify the causative pathogen promptly, particularly in patients previously exposed to antibiotics. Multiplex molecular platforms, such as the BioFire FilmArray^®^ Pneumonia Panel Plus (FAPP), allow rapid detection of multiple respiratory pathogens and resistance markers, potentially improving early therapeutic decision-making. The objective of this work is to evaluate the impact of implementing FAPP on antimicrobial therapeutic decisions in critically ill patients with suspected respiratory infection. **Methods:** We conducted a retrospective cohort study in two mixed ICUs between 2023 and 2024. All respiratory samples in which FAPP was requested were analyzed. The results were compared with conventional cultures, and changes in antimicrobial therapy following the FAPP results were assessed, classified as escalation/initiation or de-escalation/discontinuation. Concordance between FAPP and culture was evaluated, and clinical and demographic variables were analyzed. Differences between groups were assessed using *p*-values obtained from the chi-square test or the Mann–Whitney test. **Results:** A total of 363 respiratory samples were included, 88.4% from mechanically ventilated patients. FAPP was positive in 65.3% of samples, whereas cultures were positive in 23.1%. Overall concordance between FAPP and culture was 57.3%. In 42.4% of cases, pathogens were detected exclusively by FAPP. Antimicrobial therapy was modified in 29.8% of patients, predominantly through de-escalation or discontinuation (69.4% of changes). Therapeutic modifications were more frequent in nosocomial infections and in patients with a positive FAPP result. **Conclusions:** The use of FAPP in critically ill patients with suspected respiratory infection provides rapid microbiological information that significantly influences antimicrobial decision-making, particularly by facilitating antibiotic de-escalation. Although discrepancies with conventional cultures remain and require careful clinical interpretation, FAPP represents a valuable tool for antimicrobial stewardship in the ICU setting.

## 1. Introduction

Respiratory infections in critically ill patients—encompassing community-acquired pneumonia (CAP), hospital-acquired pneumonia (HAP), and ventilator-associated pneumonia (VAP)—represent a major challenge in intensive care units (ICUs) and are associated with substantial morbidity and mortality [1,2,3,4,5,6]. In these clinical scenarios, severity is typically driven by profound hypoxemia or a dysregulated immune response manifesting as sepsis or septic shock, both of which are frequently exacerbated by the patients’ underlying comorbidities [2,3].

Despite advances in diagnostic and therapeutic strategies, achieving an etiological diagnosis in pneumonia remains difficult, particularly in the critically ill. Traditional microbiological methods identify the causative pathogen in only slightly more than 50% of cases [7,8]. In HAP and VAP, prior antibiotic exposure further complicates microbiological isolation. Furthermore, the involvement of viral etiologies or multidrug-resistant (MDR) bacteria can lead to therapeutic delays and poorer short- and long-term prognoses. Such diagnostic uncertainty often results in the prolonged use of broad-spectrum empirical antibiotics [9], which fuels the cycle of antimicrobial resistance with detrimental consequences for both individual patients and public health. Additionally, atypical pathogens—responsible for over 20% of CAP cases—remain notoriously difficult to recover using standard culture media [8,10].

In recent years, significant progress has been made in microbiological diagnostics through multiplex molecular detection platforms. These systems can rapidly and simultaneously identify multiple pathogens in a single specimen. Utilizing nucleic acid amplification via real-time polymerase chain reaction (PCR), these tools have become essential complements to conventional methods. While they possess certain limitations, they have demonstrated superior sensitivity in several aspects [11,12,13,14]. Their implementation facilitates the early identification of respiratory pathogens and their associated resistance profiles, which is paramount for guiding timely and appropriate antimicrobial therapy in the ICU [11,15,16].

One such platform is the BIOFIRE^®^ Pneumonia Plus Panel (FAPP), which detects 34 targets from lower respiratory tract samples (Table 1).

The objective of this study is to evaluate the impact of FAPP implementation on therapeutic decision-making in critically ill patients with suspected respiratory infection.

## 2. Results

Three hundred and sixty-three respiratory samples were analyzed from 261 patients admitted to the ICU with clinical suspicion of respiratory infection or with unclear origin. 321 samples (88.4%) were obtained from patients on invasive mechanical ventilation, and the remainder were sputum samples. In 183 cases (50.4%), nosocomial infection was suspected.

Of the total, 237 FAPP samples (65.3%) were positive, with a 35% agreement with the positive culture results. Based on the analysis of Table 2, it is observed that the agreement between FAPP and culture for the positive and negative results was 57.3%. In 64.7% of cases, pathogens were detected that would not have been detected by conventional methods; that is, the FAPP test was positive while the culture was negative. A single case was observed in which the FAPP was negative, and the culture was positive (*Haemophilus influenzae* being isolated), considering only the microorganisms included in the panel’s spectrum. FAPP showed a sensitivity of 98.8% and a specificity of 44.8% when compared with culture as the reference standard. The positive and negative predictive values were 35.0% and 99.2%, respectively. Overall agreement between both methods was fair, with Cohen’s kappa coefficient of 0.27.

After the FAPP results were available, therapeutic adjustments were made in 29.8% of cases, with 69.4% of these adjustments involving de-escalation or discontinuation of antibiotics and 30.5% involving escalation or initiation of antibiotics (Table 3). It was observed that 97% of changes classified as positive had a positive FAPP result.

FAPP positivity was significantly higher in patients with suspected respiratory infection compared to those with signs of infection without a clear focus (Table 4). Similarly, although not statistically significant, antibiotic de-escalation or discontinuation was observed in a majority of patients with suspected respiratory infection and a negative FAAP result, compared to those in whom pneumonia was not evident.

As shown in Table 5, in cases where a pathogen was identified solely by FAAP—that is, with negative cultures—antibiotic therapy was also modified in 27.9% of cases.

In the study population, greater adjustment of antibiotic therapy was observed in nosocomial samples. Likewise, a higher incidence of nosocomial infections has been observed in surgical and trauma patients. The most frequently detected bacteria were *Haemophilus influenzae*, *Staphylococcus aureus*, and *Streptococcus pneumoniae* (Table 6). Agreement between the FAPP and culture results was higher for Gram-negative organisms (16 of 28 FAPP-positive cases confirmed by culture, 57%) than for Gram-positive organisms (10 of 27 FAPP-positive cases confirmed by culture, 37%).

## 3. Discussion

The results of our study reinforce the role of molecular diagnostics, such as FAPP, in managing respiratory infections in critically ill patients—particularly those with septic shock, those requiring mechanical ventilation, or those with suspected nosocomial infections, where early and appropriate antibiotic therapy is paramount. The superior microbiological yield and rapid turnaround times of multiplex PCR in lower respiratory tract samples facilitate earlier, targeted adjustments to antimicrobial therapy, thereby reducing both the duration of inappropriate antibiotic use and the overall course of treatment [17,18,19].

In our series, lower agreement between FAPP and culture was observed in sputum samples (43%) compared with invasive samples from patients with VAP (52.5%). Current literature continues to debate whether microorganisms detected only by FAPP represent active infection, colonization, or contamination, as PCR can identify genetic material from non-viable organisms or low-load pathogens, especially in patients with prior antibiotic exposure or low-quality samples. Consequently, there is a risk of misinterpreting these as false positives, particularly with common respiratory commensals. This highlights the importance of integrating molecular results with clinical assessments and complementary biomarkers. Conversely, discordance may stem from the inherent limitations of culture—such as low sensitivity, antibiotic-driven inhibition, or fastidious pathogens—suggesting that both methods should be viewed as complementary rather than mutually exclusive. Yoo et al. demonstrated that while sensitivity in sputum remains high (≈95–98%), specificity is lower (≈70–75%) due to additional detections not confirmed by culture. Furthermore, other authors [20,21] have highlighted the high negative predictive value (NPV) of FAPP in sputum, suggesting its utility in ruling out bacterial infection while providing the added benefit of detecting viruses and atypical pathogens.

The high proportion of positive samples obtained with FAPP compared with conventional culture aligns with previous reports that have emphasized the superior sensitivity of molecular methods for detecting respiratory pathogens [20,22,23]. This finding carries significant clinical relevance, as the information provided by the technique led to antibiotic therapy adjustments—primarily de-escalations or discontinuations—in nearly one-third of patients, underscoring its potential for optimizing antimicrobial stewardship. Nonetheless, the low concordance with culture-based methods raises questions regarding the interpretation of the positive results exclusive to FAPP. This remains an unresolved challenge [24,25,26,27], necessitating further studies to validate and contextualize these findings within routine clinical practice.

This uncertainty remains one of the primary arguments cited by microbiology laboratories to restrict the routine implementation of this technique, alongside its non-negligible economic cost. Many authors, however, contend that in advanced healthcare systems such as those in Europe, the economic impact should be evaluated in relation to the potential benefits derived from reduced morbidity and mortality—outcomes achieved by treating respiratory infections in critically ill patients both appropriately and early [28,29,30].

Culture confirmation of FAPP-detected pathogens was more frequent for Gram-negative bacteria than for Gram-positive organisms, suggesting that the lower overall positive predictive value of the panel may be driven in part by reduced culture yield for Gram-positive pathogens—potentially reflecting greater susceptibility to prior antibiotic exposure or higher rates of colonization in this group.

The FAPP’s capacity to guide antimicrobial modifications, primarily through de-escalation or discontinuation, represents a highly significant finding within the framework of antimicrobial stewardship programs. Reducing unnecessary treatments not only mitigates toxicity and associated costs but also helps curtail the selective pressure that favors the emergence of multidrug-resistant (MDR) bacteria, one of the foremost threats to global public health today [17,18,31]. Nevertheless, the challenge lies in defining clinical algorithms that effectively integrate molecular results into decision-making. This requires a balanced approach to avoid both the overinterpretation of findings—which may reflect colonization rather than active infection—and the underutilization of data that could significantly improve the prognosis of critically ill patients [4,6,31].

In our cohort, more than half of the samples originated from nosocomial infections, with a higher incidence observed in surgical and trauma patients. This finding is consistent with previous studies identifying these groups as particularly vulnerable to colonization and infection by pathogens associated with prolonged hospital stays and invasive procedures [32,33,34,35].

While other studies have reported similar results and proposed action algorithms, it is essential to emphasize the role of local epidemiology. In settings with a low prevalence of MDR bacteria, the impact of antibiotic de-escalation may be less pronounced, as narrower-spectrum agents are commonly used empirically [36]. Conversely, in environments with a high prevalence of MDR pathogens and frequent use of broad-spectrum antibiotics, FAPP-guided de-escalation holds greater potential to reduce unnecessary antibiotic exposure, minimize toxicity, and limit the further emergence of resistance [37].

This study has several limitations that warrant consideration. First, its retrospective design and the fact that it was conducted at only two centers sharing a single microbiology laboratory may limit the generalizability of our findings; epidemiological profiles and antimicrobial resistance patterns can vary substantially across different institutions and geographic regions. Second, the low concordance between FAPP and conventional culture raises questions regarding the specificity of the molecular technique and its ability to distinguish between colonization and active infection—a critical factor in avoiding inappropriate therapeutic decisions. The limited sample size of our cohort represents an important methodological constraint, as it precluded a reliable pathogen-specific assessment of FAPP sensitivity and specificity. Subgroup analyses stratified by individual microorganism or pathogen category would have been statistically underpowered and risked producing misleading estimates. The absence of a multivariate analysis adjusting for potential confounders—such as disease severity, prior antibiotic exposure, or immunosuppression—limits our ability to isolate the independent contribution of FAPP results to therapeutic decision-making. Given the sample size of our cohort, such an analysis would have been prone to overfitting and would not have yielded reliable estimates. Finally, although we observed a positive impact on the optimization of antibiotic treatment, hard clinical outcomes—such as mortality, ICU length of stay, or total antibiotic consumption—were not systematically evaluated. Future research, ideally multicenter and prospective in nature, is needed to address these limitations and further explore the real-world impact of integrating molecular techniques into diagnostic and treatment algorithms for critically ill patients with suspected respiratory infections.

In conclusion, our results support the integration of rapid molecular techniques, such as FAPP, into ICU clinical practice to enhance the diagnosis of respiratory infections. The capacity to rapidly identify a broad spectrum of pathogens and guide timely therapeutic adjustments offers a potential dual benefit: optimizing individual patient care through precise antibiotic selection and contributing to public health efforts to curb the emergence of resistance. Although challenges remain regarding result interpretation and the validation of clinical outcomes, the available evidence suggests that incorporating these tools into management protocols could represent a paradigm shift in the approach to pneumonia in the critically ill.

## 4. Materials and Methods

A retrospective cohort study was conducted between 2023 and 2024 across two multidisciplinary Intensive Care Units (ICUs) in Lleida, Spain, with a combined capacity of 30 beds. Both units are served by a single, 24 h microbiology laboratory. All clinical cases in which the FilmArray Pneumonia Panel (FAPP) and concurrent respiratory sample cultures were requested were analyzed, evaluating both the microbiological results and the subsequent therapeutic management. The FAPP was requested based on clinical judgment, either for suspected respiratory infection or as screening for fever of unknown origin. Respiratory specimens were obtained via both invasive (bronchoaspirate [BAS] or bronchoalveolar lavage [BAL]) and non-invasive techniques (tracheal aspirate [TA] and sputum).

The FAPP test was performed according to the manufacturer’s instructions and analyzed qualitatively, with the results classified as “detected” or “not detected”. For conventional microbiology, respiratory samples were inoculated on chocolate agar, blood agar, and MacConkey agar (bioMérieux, Marcy-l’Étoile, France) and incubated for 72 h at 37 ± 1 °C in a 5–10% CO_2_ atmosphere. Bacterial identification was performed using Matrix-Assisted Laser Desorption/Ionization Time-of-Flight mass spectrometry (MALDI-TOF, Bruker Daltonics, Bremen, Germany).

Patients were categorized as medical, surgical, or trauma based on their primary diagnosis at ICU admission. Demographic and clinical data were collected, including age, sex, requirement for invasive mechanical ventilation, and the origin of infection. Infection was classified as nosocomial (onset > 48 h after hospital admission) or community-acquired.

Changes in therapeutic management prompted by the FAPP results were analyzed and classified as “positive” (initiating or broadening antibiotic therapy) or “negative” (narrowing or stopping antibiotics). Concordance with lower respiratory tract cultures from the same specimen was assessed. For comparative purposes, the culture results were considered positive if they yielded any microorganism included in the FAPP target list. Therapeutic adjustments were specifically cross-analyzed with the timing of FAPP reports and the patient’s prior clinical status. This ensured that the documented impact was directly attributable to the molecular results, thereby minimizing potential overestimation of the panel’s clinical utility.

Group differences based on clinical characteristics and microbiological results were evaluated using the chi-squared test or the Mann–Whitney U test, as appropriate. For the bacterial pathogens included in the FAPP, sensitivity, specificity, and 95% confidence intervals (CI) were calculated using conventional culture as the reference method. Statistical significance was defined as a *p*-value < 0.05.

The degree of agreement between FAPP and culture was assessed using Cohen’s kappa (κ) coefficient, with the following categories of agreement: poor (<0.00), slight (0.00–0.20), fair (0.21–0.40), moderate (0.41–0.60), substantial (0.61–0.80), and almost perfect (0.81–1.00), as described by Landis and Koch [38] (statistical tool available at http://vassarstats.net/clin1.html, accessed on: 18 May 2025).

This study was conducted in accordance with the Declaration of Helsinki. Since this was a retrospective cohort study using de-identified administrative data, it was considered exempt from formal review. Patient confidentiality was maintained throughout the study, and the need for informed consent was waived.

## 5. Conclusions

Rapid molecular techniques such as FAPP emerge as effective tools for the rapid identification of respiratory pathogens in critically ill patients with suspected respiratory infection, enabling early and targeted adjustments to antibiotic therapy. In our cohort, the FAPP results led to changes in antibiotic therapy in approximately one-third of patients, with de-escalation or discontinuation of antibiotics predominating, especially in nosocomial infections and in cases where suspected respiratory infection was the guiding symptom. In more than 40% of samples, a positive FAPP result was obtained in the setting of a negative culture, and antibiotic therapy was adjusted in approximately 30% of those cases. These findings suggest a potential role for FAPP in optimizing antimicrobial use and limiting the emergence of bacterial resistance.

However, these results must be interpreted with caution. A critical limitation of FAPP and other syndromic molecular panels is their inability to distinguish between true infection and colonization. Consequently, antibiotic adjustments driven solely by FAPP positivity, without integration of clinical, radiological, and microbiological context, risk unnecessary escalation of therapy and may paradoxically contribute to the selective pressure they aim to reduce. Clinical judgment remains essential in interpreting molecular results, and positive findings should always be evaluated in conjunction with the overall clinical picture before therapeutic decisions are made.

## Figures and Tables

**Table 1:** Targets of the BIOFIRE FilmArray^®^ Pneumonia Panel Plus (FAPP).

Category (Result Type)	Target
*Viruses*	Adenovirus
	Coronavirus
	Human metapneumovirus
	Human rhinovirus/enterovirus
	Influenza A virus
	Influenza B virus
	Parainfluenza virus
	Respiratory syncytial virus
	Bacteria (qualitative result)
*Bacteria*	*Chlamydia pneumoniae*
	*Legionella pneumophila*
	*Mycoplasma pneumoniae*
	*Acinetobacter calcoaceticus*-*A. baumannii* complex
	*Enterobacter cloacae* complex
	*Escherichia coli*
	*Haemophilus influenzae*
	*Klebsiella aerogenes*
	*Klebsiella oxytoca*
	*Klebsiella pneumoniae* group
	*Moraxella catarrhalis*
	*Proteus* spp.
	*Pseudomonas aeruginosa*
	*Serratia marcescens*
	*Staphylococcus aureus*
	*Streptococcus agalactiae*
	*Streptococcus pneumoniae*
	*Streptococcus pyogenes*
*Antimicrobial resistance markers* (*qualitative, conditionally reported*)	
*Carbapenemases*	KPC, NMD, IMP, VIM, OXA-48-like
*Extended-spectrum beta-lactamases*	CTX-M
*Methicillin resistance genes*	*mecA*/*mecC* and MREJ

Source: Data on File at BioFire^®^ Diagnostics.

**Table 2:** Demographic characteristics of respiratory samples analyzed with the BioFire FilmArray^®^ Pneumonia Panel Plus (FAPP), according to community-acquired or nosocomial origin.

Variable	All Samples n = 363	Community-Acquired n = 180	Nosocomial n = 183	*p*-Value
**Age (years)**	58.5 ± 16	59.0 ± 17	58.0 ± 15	*0.367*
**Sex (male)**	269 (74.1)	126 (70.0)	143 (78.1)	*0.077*
**Mechanical Ventilation**	321 (74.1)	152 (85.0)	168 (91.8)	*0.043*
**Type of Admission**				*<0.001*
Medical	271 (74.7)	153 (85.0)	118 (64.6)	
Surgical	59 (16.3)	19 (10.6)	40 (21.9)	
Trauma	33 (9.1)	8 (4.4)	25 (13.7)	
**Respiratory Guide Symptoms**	183 (50.4)	121 (67.2)	62 (33.9)	*<0.001*
**FAPP +**	237 (50.4)	128 (71.1)	109 (59.6)	*0.007*
**Culture +**	84 (23.1)	48 (26.7)	36 (19.7)	*0.114*
**Antibiotic Change**	108 (29.8)	46 (25.6)	62 (33.9)	*0.083*
**Type of Change**				*0.235*
No Change	254 (70.2)	133 (74.3)	121 (66.1)	
Positive	33 (9.1)	14 (7.8)	19 (10.4)	
Negative	75 (20.7)	32 (17.9)	43 (23.5)	
				*0.069*
**Group (Culture/FAPP)**				
+/+	83 (22.9)	47 (26.1)	36 (19.7)	
+/−	1 (0.3)	1 (0.6)	0 (0.0)	
−/+	154 (42.4)	81 (45.0)	73 (39.9)	
−/−	125 (34.4)	51 (28.3)	74 (40.4)	

Values are expressed as percentages or mean ± (standard deviation). *p*-value: calculated using the chi-square test or the Mann–Whitney test. Positive change: initiation or escalation of antibiotic therapy; negative change: de-escalation or discontinuation of antibiotic therapy. +: positive value. −: negative value.

**Table 3:** Demographic characteristics of respiratory samples analyzed with the BioFire FilmArray^®^ Pneumonia Panel Plus (FAPP), with antibiotic treatment decision-making (n = 108), according to type of decision.

Variable	Positive n = 33	Negative n = 75	*p*-Value
**Age (years)**	54.6 ± 19	59.5 ± 15	*0.224*
**Sex (male)**	29 (87.9)	59 (78.7)	*0.256*
**Mechanical Ventilation**	32 (97.0)	70 (93.3)	*0.447*
**Type of Admission**			*0.095*
Medical	20 (60.6)	59 (78.7)	
Surgical	6 (18.2)	10 (13.3)	
Trauma	7 (21.2)	6 (8.0)	
**Respiratory Guide Symptoms**	13 (39.4)	38 (50.7)	*0.280*
**FAPP +**	32 (97.0)	42 (56.0)	*<0.001*
**Culture +**	16 (48.5)	15 (20.0)	*0.003*
**Group (Culture/FAPP)**			
+/+	16 (48.5)	15 (20.0)	
−/+	16 (48.5)	27 (36.0)	
−/−	1 (3.0)	33 (44.0)	

Values are expressed as percentages or mean ± (standard deviation). *p*-values were calculated using the chi-square test or the Mann–Whitney test. +: positive value. −: negative value.

**Table 4:** Demographic characteristics of patients with respiratory failure whose samples were analyzed with the BioFire FilmArray^®^ Pneumonia Panel Plus (FAPP) (n = 363), with and without suspected respiratory infection.

Variable	No Respiratory Infection Suspected n = 180	Respiratory Infection Suspected n = 183	*p*-Value
**Age (years)**	57.3 ± 16	59.7 ± 16	*0.065*
**Sex (male)**	142 (78.9)	127 (69.4)	*0.039*
**Mechanical Ventilation**	173 (96.1)	148 (80.9)	*<0.001*
**Type of Admission**			*<0.001*
Medical	89 (49.4)	182 (99.5)	
Surgical	58 (32.2)	1 (0.5)	
Trauma	33 (18.3)	0 (0.0)	
**FAPP +**	103 (57.2)	134 (73.2)	*0.001*
**Culture +**	42 (23.3)	42 (23.0)	*0.931*
**Antibiotic Change**	57 (31.7)	51 (27.9)	*0.429*
**Type of Change**			*0.419*
No Change	123 (68.3)	131 (72.0)	
Positive	20 (11.1)	13 (7.1)	
Negative	37 (20.6)	38 (20.9)	
			*0.002*
**Group (Culture/FAPP)**			
+/+	42 (23.3)	41 (22.4)	
+/−	0 (0.0)	1 (0.5)	
−/+	61 (33.9)	93 (50.8)	
−/−	77 (42.8)	48 (26.2)	

Values are expressed as percentages or mean ± (standard deviation). *p*-values were calculated using the chi-square test or the Mann–Whitney test. +: positive value. −: negative value.

**Table 5:** Demographic characteristics of respiratory samples analyzed with the BioFire FilmArray^®^ Pneumonia Panel Plus (FAPP) (n = 362), according to the culture/FAPP concordance group. The (+/−) result has been excluded.

Variable	+/+ n = 83	−/+ n = 154	−/− n = 125	*p*-Value
**Age (years)**	58.1 ± 16	57.4 ± 17	60.0 ± 14	*0.517*
**Sex (male)**	56 (67.5%)	111 (72.1%)	101 (80.8%)	*0.076*
**Mechanical Ventilation**	75 (90.4%)	130 (84.4%)	115 (92.0%)	*0.118*
**Sputum** **Type of Admission**	8 (9.6%)	24 (15.6%)	10 (8%)	*0.278*
Medical	61 (73.5)	122 (79.2)	87 (69.6)	
Surgical	14 (16.9)	18 (11.7)	27 (21.6)	
Trauma	8 (9.6)	14 (9.1)	11 (8.8)	
**Respiratory Guide Symptoms**	41 (49.4)	93 (60.4)	48 (38.4)	*<0.001*
**Antibiotic Change**	31 (37.3)	43 (27.9)	34 (27.2)	*0.232*
**Type of Change**				*<0.001*
No Change	52 (62.7)	111 (72.1)	90 (72.6)	
Positive	16 (19.3)	16 (10.4)	1 (0.8)	
Negative	15 (18.1)	27 (17.5)	33 (26.6)	

Values are expressed as percentages or mean ± (standard deviation). *p*-values were calculated using the chi-square test or the Kruskal–Wallis test. +: positive value. −: negative value.

**Table 6:** Distribution and frequency of pathogens detected by the FilmArray Pneumonia Panel (FAPP).

Pathogen	Frecuency (n)	Percentage (%)
*Haemophilus influenzae*	67	18.5
*Staphylococcus aureus*	44	12.1
*Streptococcus pneumoniae*	39	10.7
*Pseudomonas aeruginosa*	24	6.6
*Escherichia coli*	20	5.5
*Klebsiella pneumoniae* group	17	4.7
*Serratia marcescens*	12	3.3
*Mycoplasma pneumoniae*	12	3.3
*Streptococcus agalactiae*	9	2.5
*Enterobacter cloacae* complex	9	2.5
*Moraxella catarrhalis*	7	1.9
*Klebsiella oxytoca*	7	1.9
*Legionella pneumophila*	7	1.9
*Proteus* spp.	5	1.4
*Streptococcus pyogenes*	2	0.6
*Chlamydia pneumoniae*	2	0.6
*Klebsiella aerogenes*	1	0.3
Acinetobacter baumannii complex	1	0.3
Influenza virus A and B	27	7.4
Coronavirus	27	7.4
VRS	2	0.6
Other virus	52	14.3

Note: Data are presented as absolute frequency (n) and percentage (%) of the total number of positive detections. Percentages are calculated based on the total count of identified targets (n = 363). The FAPP allows for the detection of multiple pathogens in a single respiratory specimen.

## Data Availability

The original contributions presented in this study are included in the article. Further inquiries can be directed to the corresponding author(s).
